# The Potential Benefits of Dance Movement Therapy in Improving Couple Relations of Individuals Diagnosed With Autism Spectrum Disorder—A Review

**DOI:** 10.3389/fpsyg.2021.619936

**Published:** 2021-02-18

**Authors:** Einat Shuper Engelhard, Maya Vulcan

**Affiliations:** ^1^Faculty of Humanities and Social Sciences, Graduate School of Creative Art Therapies, Kibbutzim College of Education, Tel Aviv, Israel; ^2^Faculty of Social Welfare and Health Sciences, Graduate School of Creative Art Therapies, Emili Sagol Creative Arts Therapies Research Center, University of Haifa, Haifa, Israel

**Keywords:** autism spectrum disorder, couple therapy, dance movement therapy, dance movement therapy for couples, mirroring

## Abstract

A review of current literature indicates that adults diagnosed with autism spectrum disorder (ASD) feel the need for intimate and sexual relationships and maintain such relationships despite and alongside their difficulties in emotional communication, social interactions, reciprocity, and verbal and non-verbal expression. This understanding calls for the development of intervention programs designed to support the specific needs and address the problems of couples where one partner is diagnosed with ASD. In view of the relevance and significant part played by body and movement in emotional development and psychotherapy, the present article offers a review of studies examining the contribution of dance movement therapy to both the quality of life and functioning of adults with ASD and therapeutic processes in couple therapy. This review aims to establish an infrastructure for the construction of intervention programs and for future studies designed to enhance the quality of life and independence of adults with ASD.

The development of adult intimate relationships and the transition into couplehood are part of most people's life cycles, but these transitions become very challenging for individuals diagnosed with autism spectrum disorder (ASD) in as much as social interactions, emotional communication, and reciprocity, which are essential for interpersonal relationships (Le et al., 2010), are made more difficult due to the condition itself (Orsmond et al., 2004).

Research on couple therapy for adults diagnosed with ASD is in its infancy, and the core issues of the studies relate to the need for a better understanding of the unique aspects of intimacy and sexuality for these clients. Studies have examined the prevalence of marital relationships, reporting that 16% of individuals diagnosed with ASD get married (Tani et al., 2012). However, the specific difficulties entailed in the condition are an obstacle to the maintenance of a steady, long-term relationship, and significant support is needed to assist the development of these relationships and, in particular, in preserving them (Seltzer et al., 2004; Schöttle et al., 2017; Strunz et al., 2017; Dembosky, 2020).

Current research also explores the experience of typically developed (TD) individuals who are married to partners with ASD. Women married to partners with ASD describe their subjective experience of struggling and loneliness, which all-too-often lead to crises after marriage (Deguchi and Asakura, 2018), and the most problematic areas involve issues of affective communication, problem-solving communication, and quarreling (Roy and Dillo, 2017). Another complexity entailed in these relationships relates to the existence of social stigmas that partners may experience (Naoko et al., 2020).

A recent study which examined the perceptions of adults with and without ASD regarding intimacy found that, among the two groups, intimacy included communication, sharing and similarity, respect and safety for self and other, and working on the relationship. Barriers for both groups included intra- and interpersonal conflicts; participants with ASD specifically highlighted uncertainty about relationships and communication, feeling that they did not have the required knowledge and skills. These findings suggest that individuals with ASD and TD individuals have similar notions of intimacy, yet they have different challenges in experiencing it (Sala et al., 2020).

An additional study (Cheak-Zamora et al., 2019) explored the sex and relationship experiences of 27 adults with ASD. The study found that the participants craved a relationship, but only few reported having partners. Among those that did, their actual relationships rarely met ideals. All of the participants expressed the need for tutoring and coaching that cover basic intimacy topics. These studies point to gaps between the desired and the actual life in the realm of intimacy and sexuality for adults with ASD. This gap highlights the need for further studies which would examine specifically tailored intervention programs intended to provide support for marital and sexual issues with this client population.

In spite of the recognition of the particular difficulties described above, the literature survey indicated that there is a distinctive lack of empirical studies that examine interventions for improving couple relationships in individuals with ASD. It is possible that one of the reasons for this lack is the fundamental question as to the efficacy of exclusively verbal work (Koegel et al., 2009; Anderberg et al., 2017) in as much as the clients' communication issues may pose significant problems for reciprocal verbal engagement and emotional reciprocity (Naber et al., 2008; Baron-Cohen, 2010; Eigsti et al., 2011; Vulcan, 2016).

At this point, in view of these lacunae, the manifested need at the clinical level, and the critical role of the body in understanding the complexity of an adult with ASD, we propose to take a step toward addressing these issues by offering a review of studies examining the advantages of dance movement therapy (DMT) to both the quality of life of adults with ASD and processes of couple therapy.We suggest that a specific focus on these two lines of research into ostensibly different “bodies of knowledge,” as offered in this review, constitutes a step toward a research protocol and effective clinical interventions that would support the formation and maintenance of intimate relationships of adults with ASD.

This review was conducted in accordance with the PRISMA Checklist (PRISMA, 2015). The following three criteria were used to select the research studies for this review: (a) use of specific key terms (“DMT,” “Couple Therapy”, “Autism Spectrum Disorder,” “Asperger's Disorder/syndrome,” and “Pervasive Developmental Disorder”); (b) publications in international evidence-based English academic journals between 1980 and 2020, and (c) adult individuals identified as having ASD, Asperger's, or pervasive developmental disorder who were involved in DMT and adult individuals in a romantic relationship who took part in DMT-C. The following electronic databases were used to conduct this research study: Medline, Pubmed, Cinahl, and Springer Link.

## ASD and DMT

DMT is based on the assumption that pre-verbal, pre-mentalized bodily memories play an important role in relationships and influence our intimate experience (Shuper Engelhard, 2019b,c,d,e). DMT fosters embodied experiences through body-based interventions and movement, and words are used to increase self-recognition, self-other distinction, and self-awareness on a bodily level, which positively influence social understanding (Fuchs and Koch, 2014; Koch et al., 2015). Moreover, it has been shown that moving between actions or inner felt experiences and their verbal expressions and practicing these transitions increase the ability to communicate feelings, needs, and emotions (Devereaux, 2012).

DMT interventions include various types of non-verbal mirroring, reflection, use of rhythm, regulatory movement, and movement synchrony–all of which are broadly aimed at helping the client practice relationship skills to attain bodily attunement and empathy and to get acquainted with feelings and needs through the experience of synchronization (McGarry and Russo, 2011; Behrends et al., 2012).

Through the body, DMT addresses issues of emotion and cognition, applying empathic reflection via mirroring of the client's movements in order to form a relationship and enhance emotional understanding of self and other. The assumption underlying this therapeutic modality is that these basic, non-verbal interactions, within the safe and secure clinical setting, serve as a bridge for the formation of relationships and for the enhancement of communicative functioning in relationships outside the clinic in day-to-day context (Baron-Cohen and Wheelwright, 2004).

Studies examining the contribution of DMT with adults diagnosed with ASD have found that their ability to synchronize in open and free movement is limited compared to TD but not completely impaired, and researchers recommend using these tools to develop the social skills of empathic tendency, emotional recognition, and affective engagement (Brezis et al., 2017). Studies, respectively, show that the practice of mirroring, synchronization, rhythm, and reciprocity in movement contributes to social involvement, body awareness (DeJesus et al., 2020), emotional engagement, rapport, and intersubjective communication (Delafield-Butt et al., 2020), emotional empathy (Mastrominico et al., 2018), and the expression of emotions (Takahashi et al., 2019). Several studies in the field describe the effect of 1-h intervention per 6 weeks on regulation, imitation, and interaction (Mateos-Moreno and Atencia-Doña, 2013), for 7 weeks on improvement in psychological well-being, body awareness, self-other awareness, and social skills (Koch et al., 2015), for 8 weeks on the ability to form relationships (Edwards, 2015), and for 10 weeks on emotional expression (Hildebrandt et al., 2016), body image (Koch et al., 2016), and cognitive empathy (Koehne et al., 2016b). The interventions in all these studies are based on movement reflection, followed by a verbal discourse that allows the experience to be processed and communicated from the body to the here-and-now. The researchers in all of these studies emphasize the contribution of the practice of empathy in movement (through synchronizing and joining others) to emotional and social understanding (Table 1).

**Table 1:** Adults diagnosed with autism spectrum disorder and dance movement therapy.

**References**	**Study design**	**Objective**	**Sample**	**Intervention length**	**Intervention elements**	**Evaluation tools**	**Results and conclusions**
Brezis et al. (2017)	Experimental setup	Examining synchronization during an open-ended joint improvization paradigm—the mirror game (MG), aiming to assess whether participants with ASD are capable of attaining co-confidence and whether their MG performance relates to broader motor and social skills	Thirty-four participants with high-functioning ASD and 38 TD	The experimental sessions lasted for 2–3 h	The Mirror Game Procedure, including the reaching task, proprioception task, repetitive motion task, and following a moving target task	MG; the Revised Neurological Examination for Subtle Signs (PANESS); the Florida Apraxia Battery, an imitation battery based on Fitzpatrick et al. (2013); Toronto Empathy Questionnaire, TEQ; Reading the Mind in the Eyes, RMET; Toronto Alexithymia Scale, TAS.	ASD participants can attain moments of high motor synchronization, even during an open-ended task. Joint synchronization was not inexistent, but simply reduced, in individuals with ASD, and their existing synchronization skills may be further harnessed in clinical settings to support their personal expression and social rapport
						Following the MG, the participants completed a naturalistic conversation task	
DeJesus et al. (2020)	Systematic review	Identifying how dance promotes positive benefits for the negative symptoms in ASD	Five studies fulfilled the inclusion criteria	–	Analysis of article titles, reading summaries of articles that had been identified, reading the entire article, analyzing article references from those that had been read in their entirety	Research question based on the PICO strategy. Five databases were used: the National Library of Medicine (MEDLINE-PubMed), Science Direct, Web of Science, Scopus, and PsycINFO (APA—American Psychological Association)	Dance practice can promote a beneficial effect on the negative symptoms of individuals with ASD in daily life as well as positively influence biopsychosocial aspects. It contributes to body awareness and social involvement using techniques like mirroring, synchronization, rhythm, and reciprocity
Delafield-Butt et al. (2020)	Case study design	Examining narrative meaning-making between a nonverbal young woman with severe autism and her new therapist	One participant	Not described	Intensive interaction, which involves interacting with a person by using their own sounds and movements	Microanalysis and coding video footage	Intervention was successful in promoting emotional engagement, rapport, and intersubjective communication
Edwards (2015)	Qualitative, reflective participant observation (case study) methodology within a realism perspective	Exploring sensory experiences of adults with autism and to find out how they form relationships, including attachment behaviors, within a DMT group	Four adults diagnosed with autism	Eight-week period (the participants attended DMT sessions for 12 months prior to the research period)	Not described	(1) Movement observations by therapists, (2) verbal feedback from the participants was noted at the end of each session, and (3) the therapists' reflective journals	Accommodating and adjusting to sensory differences influenced how the participants formed relationships, which suggests that DMT was a beneficial intervention for them
Hildebrandt et al. (2016)	Cohort study RCT	Examining the effect of movement therapy intervention (based on DMT) on negative symptoms (NS) in participants with ASD	Seventy-eight individuals with ASD: 55 in the intervention group and 23 in the control group	Ten weekly sessions with a duration of 60 min	Manualized DMT intervention. Each session consisted of three mirroring exercises and one verbal processing element. The control group received no intervention	Scale for the Assessment of Negative Symptoms (SANS)	The study highlighted the influence of dance practice on negative symptoms, including emotional expression, with an interaction effect at the significance level of 0.1, indicating symptom reduction in the dance group [*F*_(1.4)_ = 2.99, *p* = 0.09]. The effect sizes were small but clinically meaningful, and the resulting patterns were in accordance with theoretical expectations.
							The effect sizes indicate a moderate effect for group and a small effect for the interaction term
Koch et al. (2015)	Cohort study, clinical trial	To examine the effectiveness of DMT intervention based on mirroring in movement with ASD in increasing body awareness, social skills, self–other distinction, empathy, and well-being	Thirty-one young adults diagnosed with ASD: 16 in the intervention group and 15 in the control group	Seven weeks of hourly sessions once a week	Structured treatment—manualized DMT intervention of mirroring exercises and verbal processing.	(1) Pretest and posttest Questionnaires—Heidelberger State Inventory, Questionnaire of Movement Therapy; (2) self–other awareness self-constructed scale; short form of the EES. FBT subscale of social skills; observations of mirroring modalities through the concept of co- and self-regulation from Eberhard-Kaechele (2009, 2012); qualitative expressive measures (painting a picture or writing a short poem)	DMT can be effective and feasible for the treatment of individuals with ASD, causing improvement in psychological well-being, body awareness, self–other awareness, and social skills compared with the control group [*F*_(1.27)_ = 2.95, *p* = 0.04, *d* = 0.63]. Outcomes improved significantly in the expected direction with medium to large effects (effect sizes of 0.61–0.91)
					The control group received no intervention		
Koch et al. (2016).	Intervention research	Investigating the effects of DMT on body image in autism	Ten young adults with ASD	Ten weekly sessions of DMT	DMT intervention based on therapeutic mirroring and verbal processing	Body-image-sculpture test, a projective test (KST)	The results indicated an improvement in body image both on the individual and on intersubjective aspect in a group context.
							*N* = 64 would be needed to fulfill the requirements of power analysis given the effect sizes of the present study
Koehne et al. (2016a)	Intervention-controlled parallel-group clinical trial (proof-of-concept study)	Establishing the efficacy of imitation- and synchronization-based DMT intervention (SIDMI) in fostering emotion inference and empathic feelings with high-functioning adults with ASD	Fifty-five adults with ASD: 29 in the intervention group and 26 in the control group	Ten weeks of a DMT intervention administered in 10 90-min sessions over the course of 3 months	DMT interventions focused on interpersonal movement imitation and synchronization (SI-DMI) and a control movement intervention focusing on individual motor coordination (CMI)	Multifaceted empathy test targeting emotion inference and empathic feelings; self-rated interpersonal reactivity index; assessment of spontaneous interaction in movement pre–post design; we proposed a conservative medium effect size of η^2^ = 0.13, resulting in a total sample size of *n* = 56, given a significance level of 5% and power of 80%	DMT focused on interpersonal imitation and synchronization changed emotional inference when compared with the control movement intervention group (*p* = 0.04, η^2^ = 0.09).
							Interaction effects related to social reciprocity were also promoted by imitation/synchronization (*p* < 0.001, *d* = 1.27) and reciprocity/dialogue (*p* = 0.04, *d* = 1.25), respectively. Those treated with SI-DMI showed a significantly larger improvement in emotion inference than those treated with CMI. SI-DMI increased synchronization skills and imitation tendencies, as well as whole-body imitation/synchronization and movement reciprocity/dialogue, compared to CMI
Koehne et al. (2016b)	Cohort study, experimental design	Investigating the effect of perceived interpersonal synchrony on cognitive and emotional empathy in individuals with and without ASD	Twenty adults with ASD and 22 NT participants	Not described	Engaging in two simple leader—follower finger tapping communication tasks	The Movie for the Assessment of Social Cognition (MASC); the revised version of the “Reading the Mind in the Eyes” task (RME)	The results point to a mediating role for interpersonal synchronization in cognitive empathy, a mechanism that seems attenuated, yet not absent, in ASD
Mastrominico et al. (2018)	Cohort study—RCT	Examining the effects of DMT on empathy for adults with ASD	Fifty-seven individuals with ASD: 35 in the intervention group and 22 in the control group	Ten DMT sessions once a week	Manualized form of DMT.	Empathy, measured with the Cognitive and Emotional Empathy Questionnaire (CEEQ); observational measures such as the SANS-scale for negative symptoms; the subscale Empathic Concern of the Interpersonal Reactivity Index (IRI)	Mirroring interventions had an effect on empathy, relationships, and perception of the feelings of others as well as psychological health. The main effect of time compared with control group yield findings for emotional empathy [*F*_(1,55)_ = 12.55, *p* = 0.001, η^2^ = 0.19) and its subscales mirroring [*F*_(1,55)_ = 9.22, *p* = 0.004, η^2^ = 0.14) and empathic concern [*F*_(1,55)_ = 4.99, *p* = 0.030, η^2^ = 0.08]
					Every dance movement session was built from the same elements, including a warm-up, two different mirroring exercises, and a final verbal part		
Mateos-Moreno and Atencia-Doña, 2013	Experimental study	Assessing the effectiveness of DMT and music therapy (MT) procedures on adults with severe autism	Sixteen participants	Thirty-six 1-h sessions during 17 weeks	A number of varied MT and DMT activities in each session. The core of each session consisted of dance, instrumental practice, singing, and observation/mimicking of movement	Revised Clinical Scale for the Evaluation of Autistic Behavior (ECA-R). During the treatment, eight measurements were taken (one every 3 weeks)	The observed power by partial squared Eta was high (2 = 0.90, *p* < 0.001) in the progression of measures in the experimental group.
							The value of partial squared omega ω^2^ = 0.69 estimates an equally high effect size (Cohen, 1988) of the treatment if systematically applied to the severely affected adult autistic population. The observed power (Cohen's *d* = 1.18, *r* = 0.51) suggests a moderate effect size (Cohen, 1988) on the interaction disorder.
							The observed power (Cohen's *d* = 1.88, *r* = 0.68) suggests an elevated effect size (Cohen, 1988) on the instinct disorder.
							The study provides preliminary evidence for the high benefits of jointly using MT and DMT with severely affected autistic adults
Takahashi et al. (2019)	Systematic review	To verify the quality of DMT and ASD studies using Preferred Reporting Items for Systematic Reviews and Meta-Analyses (PRISMA) guidelines and to evaluate the effectiveness of DMT interventions for individuals with ASD	Seven studies	Not described	The criteria were (a) use of specific key terms, (b) publications in international evidence-based English academic journals between 1970 and 2018, (c) the specific words were used in the papers' abstract, and (d) individuals involved in DMT were identified as having ASD, Asperger's, or PDD	Keyword analyses of four electronic databases—Medline, Pubmed, Cinahl, and Springer Link	Imitation (mirroring) interventions helped individuals with ASD improve their social skills. DMT treatment will help mitigate the difficulty of sociality in ASD and is an effective preventive or treatment measure for improving social skills
Wadsworth and Hackett (2014)	An observational single-case study and a practice-based research approach	Introducing a structured narrative approach in the form of the six-part story within DMT with an adult with ASD	One participant	Seven DMT sessions lasting 45 min	A consistent format including a warm-up, mirroring, a “six-part story,” and relaxation techniques	The BASIC-Ph; the creative arts therapies session rating scale (CAT-SRS); Picture Communication Symbols TM Boardmaker program	The study results indicate that DMT practice can incorporate structured narrative approaches in work with adults with ASD
Zapata-Fonseca et al. (2019)	A quantitative description	Providing a quantifiable link between individual motor movement markers as potential diagnostic tools for ASD and social interaction deficits of ASD, which are currently the two main diagnostic criteria	Ten dyads of adult participants	Not described	Interpersonal coordination complexity matching (CM), individual movement profiles, multi-scale movement variability	The time series of embodied interaction was recorded by means of a minimalistic human–computer interface paradigm that has become known as the “perceptual crossing experiment” (PCE)	An overall mutual coordination was reached at the dyad level, even in this highly constrained, minimal environment. Research supports the usage of computer-mediated and tactile interactions for understanding the relationship between movement-based coordination and social engagement in patients with ASD

Following this, our review aims to examine the theoretical basis for a DMT model that could hopefully assist adults with ASD in their intimate romantic relationships by enhancing satisfaction, working toward stabilizing the partnership, and extending its duration.

## DMT for Couples

DMT for couples (DMT-C) focuses on body sensations and movement and lays the ground for symbolic discourse, insight, and processing of psychic materials that influence the couple's relationship (Wagner and Hurst, 2018). The majority of the literature on the subject is based on case studies offering movement experiences combined with verbal discourse during the couple therapy session (Table 2).

**Table 2:** Dance movement therapy for couples.

**References^**a**^**	**Study design**	**Objective**	**Sample**	**Intervention length**	**Intervention elements**	**Evaluation tools**	**Results/conclusions**
Cunningham (2014)	Mixed-method case study; no control group	Reflecting on the positive impact DMT has on emotional stress factors with couples experiencing infertility	Two Counselors	No description	No description	(1) Quality of life (QoL) questionnaire; (2) qualitative questionnaire	DMT allows the couple to process their traumatic experience, re-imagine themselves, and move toward the actualization of a new unmet potential
DeBoer (2006)	Qualitative research	Exploring the experience of partner dance classes as an adjunct to couple counseling	Eight couples	1 h weekly	No description	Interviews before, during, and after the class series	The couples' movement reflects content in the marital relationship
Hawkes (2003)	Clinical report	Describing the use of partner dance as a “container” for the experience and feelings of participants in a group	No description	Weekend workshops, 12 h over 2 days	Facilitating awareness of and experimenting with posture, how participants move and sense their bodies, and how they relate to others through their bodies	No evaluation tools	Dancing with a partner particularly opens up issues concerning the other
Kierr (2011)	Theoretical model	Identifying techniques which are designed to develop healthy sexuality	—	—	Here-and-now exercises, guided imagery, assertiveness training, sensory integration activities	—	—
Kim et al. (2013)	Qualitative research method	To configure and apply the kinesthetic empathy program and to assess its effectiveness for married couples in conflict	Three couples	Four 2-h sessions	Body concept—focusing on breath awareness. Space concept—focusing on personal space awareness. Effort concept—focusing on care-giving daily movement. Relationship concept—focusing on sharing most memorable moment of martial life	(1) Focus group interview and (2) semi-structured and unstructured questionnaire	Expressive movement, synchronization, and mutual attunement through movement had a positive impact on the perceived relationships, an increase of kinesthetic empathy, and an improvement of the participants' ability to emotionally attune in relation to their partners
Lacson (2020)	Theoretical model and case example	Exploring the use of mirroring with couples to foster secure attachment by means of attunement on a bodily-based level	–	–	No description	–	Paying attention to the body may be the very action that interrupts a couple's destructive dance cycle, challenges old attachment patterns, and opens the gate to more secure ways of relating to and experiencing one another
Patterson et al. (2012)	Quantitative research	Identifying specific nonverbal behaviors associated with different affective states	Thirty partners	–	No description	(1) Self-report questionnaire, (2) saliva samples, (3) a video-recall procedure rating affect during two conversations	Body movement in couple interactions can be used to identify affective states which are relevant to the partners' communication
Pietrzak et al. (2017)	Qualitative research	Fostering emotional regulation, multifaceted empathy, and conflict resolution via emotional activation therapy	Two couples diagnosed with borderline personality disorder	20 h	Imitation of a personal choreography, movement synchronization, and collaboration in resolving incidents of nonsynchronization of movement	Pre- to post-test change scores	The couples reported greater relationship satisfaction, a more securely attached relationship, and increases in empathy in the relationship
Polo (2010)	Phenomeno-logical research methods	Gaining an in-depth understanding of the various dimensions of relationship experienced by Argentine tango dancers	Three male tango “leaders” and three female “followers” of various experience levels (dance partners only)	Single 2-h session	Dancing tango socially	(1) The participants dancing tango were recorded; (2) they were interviewed separately using a customized video for each as a prompt discussion of the dance relationships	DMT could be used in tandem with tango by exploring effort and effort qualities that best support the role of the leader and the role of the follower
Wagner and Hurst (2018)	Theoretical model and case example	Development of a theoretical framework for DMT with romantic partners	One couple	–	Palm-to-Palm Work as the Dance of the Internal Family System (e.g., experiment with degrees of pressure, exploring leading and following)	–	Dance/movement therapists can recognize and process unresolved development dances that need re-patterning

a *In all studies, the intervention was performed by the researchers*.

Research in the field of DMT-C found that the experience of expressive movement, synchronization, and mutual attunement through movement had a positive impact on the perceived relationships, an increase of kinesthetic empathy, and an improvement of the participants' ability to attune in relation to their partners' needs (Kim et al., 2013). These skills are precisely those so essential in couple relationships (Le et al., 2010), which may be complex and difficult for adults diagnosed with ASD (Orsmond et al., 2004). Another study found that the imitation of a personal choreography, movement synchronization, and collaboration in resolving incidents of non-synchronization of movement resulted in higher marital satisfaction, more secure attachment, and higher empathy in the relationship (Pietrzak et al., 2017). Furthermore, couples reported experiences of movement with their hands (Wagner and Hurst, 2018), being led in space by their partner (Polo, 2010), mirroring and imitating movement (DeBoer, 2006; Patterson et al., 2012; Lacson, 2020), raised awareness of emotional content from the couples' daily lives, and the ability to understand the emotional needs of the partner. The interventions in the various studies range from 8 h (Kim et al., 2013) of practice to 12 h (Hawkes, 2003; Pietrzak et al., 2017) as similarly described above in DMT interventions with adults diagnosed with ASD.

In a recent research project, nine couples with TD participated in 12 sessions of DMT-C (Shuper Engelhard, 2018, 2019a,b,c,d,e; Shuper Engelhard and Vulcan, 2018; Vulcan and Shuper Engelhard, 2019). A total of 126 h of therapy were transcribed and coded. The session began with an invitation to observe the “spontaneous joint movement” (for example, the couple was invited to walk around the room while observing their personal pace and their dyadic pace). As the session continued, the “spontaneous joint movement” was expanded (for example, through the examination of different rhythms). This expansion was designed to meet diverse feelings and emotions evoked through different movement patterns of the partners. The following phase in the session included the “mirroring of movement,” where each partner was invited to move like his/her partner. After each experience of movement, the therapist asked the couple to practice an inner observation of feelings, emotions, memories, and images that emerge after the movement experience. At the end of the session, the couple was invited to share with each other their movement experience in words.

This study found that the participants learned new verbal and non-verbal communication skills, and as a result, they experienced a sense of improved intimacy, and their relationship became more enjoyable. It was also found that the shared body experience helped the partners to increase mutual engagement, responsiveness, and attentiveness (Shuper Engelhard, 2019c). They each learned about the needs of the other, including which bodily experiences bring about a sense of security in the relationship (Shuper Engelhard, 2019d).

In all the studies described, the couples were without any formal diagnosis, except for a single study involving couples with borderline personality disorder (Pietrzak et al., 2017). The review did not find any studies on DMT for adults diagnosed with ASD who are involved in a romantic relationship. All of the interventions offered for DMT with couples are similar to the type of interventions recommended for adults diagnosed with ASD (e.g., synchronization, mirroring, imitation, etc.) in order to enhance social skills and the ability to recognize the other's emotional expression and feelings. Moreover, both studies in DMT-C and studies of DMT for adults diagnosed with ASD suggest the combination of sensory and kinesthetic experience with verbal processing. In couple therapy, the partners reported that their communication improves as the somatic and kinetic information is translated into verbal discourse (Vulcan and Shuper Engelhard, 2019). This is compatible with Bird and Viding's model of empathy (Bird and Viding, 2014; Fletcher-Watson and Bird, 2020), as the contribution of verbalization of the sensory experiences among adults diagnosed with ASD (Hildebrandt et al., 2016) is important to the development of emotional cognition that should be based on learning the connections between emotional action and emotional experience (i.e., identifying the emotion through its expression). These insights reinforce the importance of an intervention that focuses on identifying the other's body gestures as a way to bring about more satisfying communication in the relationship.

## DMT-C for ASD

We aim to offer this review as a springboard for the development of effective intervention models, which would enhance the ability of adults with ASD to form intimate relations. Further research that can validate the use of DMT-C for ASD is warranted, but based on this review, we would recommend a research plan which, we believe, is likely to generate important and novel clinical information and help to reduce social isolation in adults with ASD. The proposed intervention protocol focuses on mirroring, synchronization, and mutual coordination. A DMT-C session for ASD should consist of a warm-up phase, followed by movement experiences and a closing verbal part. This protocol should take place for at least 6 weeks, with two 1-h sessions each week, as all of these were found to contribute to both couple therapy and interventions for adults with ASD. In this proposed study, the efficacy of DMT-C with ASD will be examined by using an RCT model comparing three groups of couples: two intervention groups [DMT-C, verbal therapy (VT), and a delayed DMT-C group (DG)]. The comparison of the intervention protocol will help determine the unique contribution of each treatment modality to improvements in the subjects' capacity for communication, intimacy, and reflective functioning, whereas the comparison of each modality with the DG is aimed to verify improvement beyond natural maturation.

Moreover, literature on music therapy and ASD is continuously growing (Thompson and Elefant, 2019; Epstein et al., 2020; Nielsen and Holck, 2020), and the benefits of DMT may partly be due to the therapeutic effects of music by itself. Perhaps future research could incorporate an experimental design in which DMT-C could be compared with a control group exposed to the same music but without the dance element to determine the effect of dance on its own. Additionally, we would propose DMT as an appropriate intervention designed to raise emotional awareness and social skills and believe that a suitable manualized intervention is likely to be helpful in developing programs for community interventions tailored to the needs of people diagnosed with ASD. By working on the quality of intersubjective communication through DMT, we expect to see an improvement of the quality of the couples' relationship, of the duration of the relationship, and in the partners' satisfaction with each other.

## Discussion

Drawing on a number of empirical studies, the aim of this review was to create a comprehensive picture of current research addressing the issues of couple relationships where one partner is diagnosed with ASD in order to understand the potential contribution of DMT in these cases. The survey suggests that DMT-C can contribute to synchronization, adjustment, and familiarity between the partners and that this form of support may be beneficial in terms of the relationship and help the partners cope with the difficulties entailed in ASD.

Both DMT interventions with adults diagnosed with ASD and DMT-C were found to be a valuable mode of supporting interpersonal communication and empathy through movement experiences and may thus be constructively combined to help adults with ASD in engaging with and maintaining intimate relations. The review shows that bodily mirroring has been extensively referenced in the context of working with clients diagnosed with ASD (Baron-Cohen, 2004; McGarry and Russo, 2011; Koehne et al., 2016b; Feniger-Schaal et al., 2018) in order to support social skills and emotional awareness.

As described previously, this is also precisely what is practiced in DMT-C when there is a disruption or difficulty in using these mechanisms. The ability to identify with one's emotion, alongside the establishment of a distinction between the needs and feelings of each one of the partners (Siegel, 2012, p. 6), is the basis for intimate relations, symbolizing the extent of the couple's closeness and the freedom to express the self fully in the relationship while enabling the partner to possess a separate self (Lerner, 2017).

This review emphasizes that heightened emotional awareness is achieved through the transition from awareness of bodily experiences to their verbalization within the DMT session. The articles reviewed emphasize that DMT, which draws attention to the bodily experience and sensations, is helpful for individuals who find it difficult to reflect verbally on their emotions, thoughts, and feelings (Shuper Engelhard, 2019e). It is important to understand the uniqueness of movement for the processing of embodied knowledge as described by Koch et al. (2015, p. 2): “the mind is not hidden but directly expressed in other persons' embodied actions' from the earliest days of infancy.” This understanding, alongside the translation of physical and emotional experiences into verbal discourse in order to improve and support intimate relationships, will aid in the development of intervention programs for adults diagnosed with ASD.

## Conclusions

The benefits of integrating movement into couple therapy and into emotional therapy for individuals with ASD are gaining more recognition and scope in research and clinical practice. This review indicates that DMT may contribute to the well-being and quality of day-to-day lives of adults with ASD. The ability to identify and express bodily sensations and to relate them to emotions may assist the partners in forming and maintaining adult intimate and sexual encounters, identifying and communicating needs, and feeling understood and loved by another. Moreover, understanding the role of the body and its movement in therapy when emotional discourse is limited can help formulate unique intervention programs for adults diagnosed with ASD as DMT can assist in improving communication and intimacy which may later lead to a higher degree of autonomy when one of the partners is diagnosed with ASD. By offering this literature review, we hope to make a contribution to a better quality of life for adults with ASD by helping them to overcome isolation and engage in meaningful and enjoyable familial and social interactions. The implications of the review will hopefully encourage researchers to take these insights further in future research.

## Author Contributions

ES and MV have contributed equally to the article and approved the submitted version. Both authors have contributed to the main body of text and to its main ideas.

## Conflict of Interest

The authors declare that the research was conducted in the absence of any commercial or financial relationships that could be construed as a potential conflict of interest.
